# Supplementary material for: The interactome of intact mitochondria by cross-linking mass spectrometry provides evidence for coexisting respiratory supercomplexes
Source: Mol Cell Proteomics. 2017 Dec 8;17(2):216–32. doi: 10.1074/mcp.RA117.000470 (PMC5795388; doi:10.1074/mcp.RA117.000470)
Supplement: Supplemental Data [file supp_RA117.000470_133922_0_supp_23978_fzffwf.zip › spectra_annotation/mito_spectra_annotation/59-1-6-1-3-1.pdf]

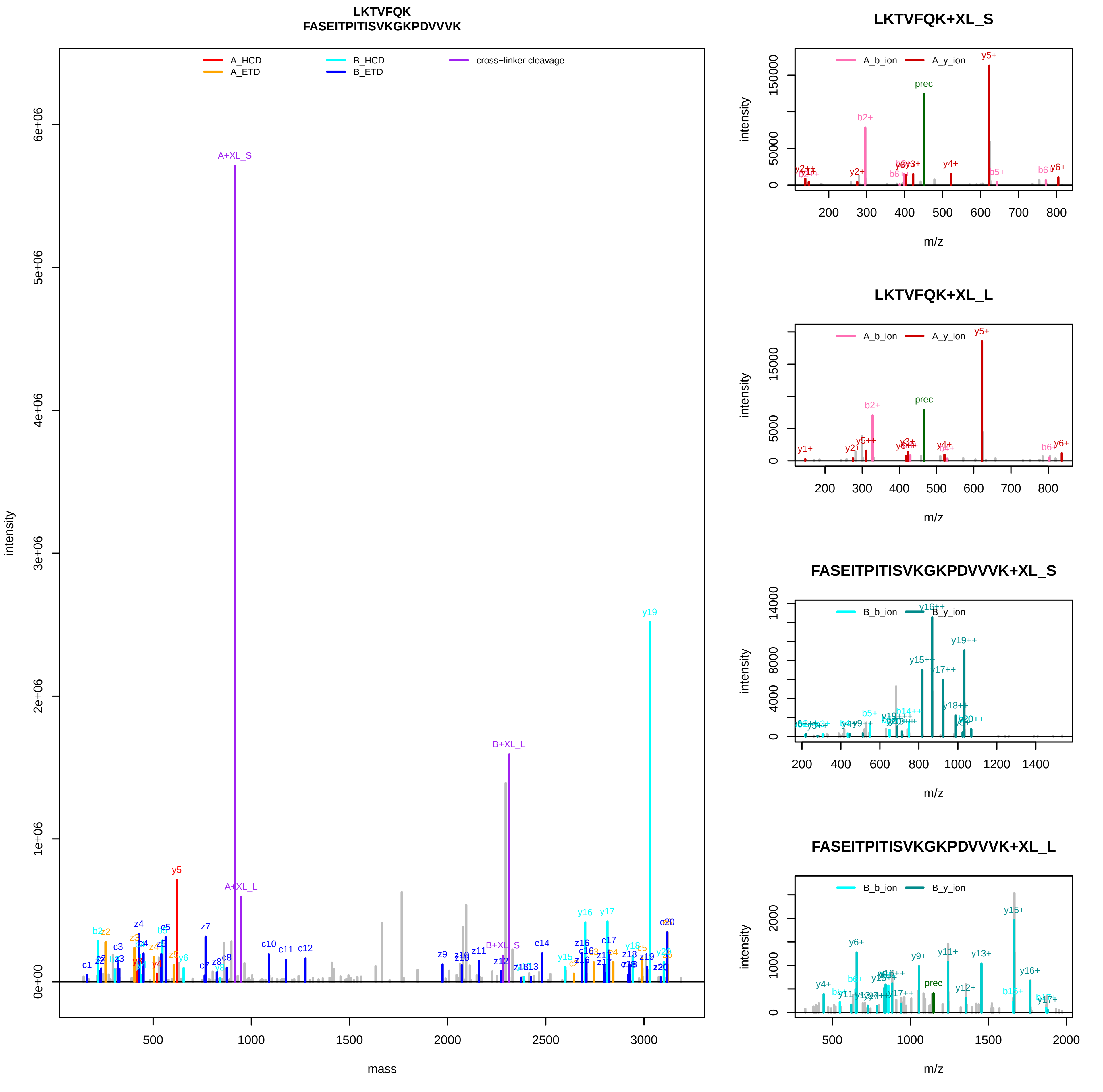
LKTVFQK
FASEITPITISVKGKPDVVVK
A_HCD
A_ETD
B_HCD
B_ETD
cross−linker cleavage
A+XL_S
A+XL_L
B+XL_S
B+XL_L
y5
y19
y16
y17
intensity
mass
0e+00
1e+06
2e+06
3e+06
4e+06
5e+06
6e+06
500
1000
1500
2000
2500
3000
LKTVFQK+XL_S
LKTVFQK+XL_L
FASEITPITISVKGKPDVVVK+XL_S
FASEITPITISVKGKPDVVVK+XL_L
A_b_ion
A_y_ion
B_b_ion
B_y_ion
prec
y5+
b2+
y4+
y6+
y16++
y19++
y15++
y17++
y18++
y20++
y15+
y6+
y9+
y11+
y13+
y16+
y12+
m/z
